# Editorial: Personalizing Treatment in IBD: Hype or Reality in 2020?

**DOI:** 10.3389/fmed.2021.673991

**Published:** 2021-05-07

**Authors:** Fernando Gomollón, Edouard Louis

**Affiliations:** ^1^Inflammatory Bowel Disease (IBD) Unit, Department of Medicine, Facultad de Medicina, Hospital Clínico Universitario “Lozano Blesa”, Instituto de Investigación Sanitaria de Aragón (IIS) Aragón, Centro de Investigación Biomédica en Red de Enfermedades Hepáticas y Digestivas (CIBEREHD), Zaragoza, Spain; ^2^Department of Gastroenterology, University and Centre Hospitalaire Univestitaire (CHU) Liège, Liège, Belgium

**Keywords:** personalized, IBD, Crohn's disease, ulcerative colitis, prediction

Let us go to the daily clinic. Ana Isabel, Raúl, and José Luis are the real names of three of IBD (inflammatory bowel disease) patients to be seen tomorrow at the office. We will share information on their symptoms, tests results, treatment plans and worries for the following months. We are confident, for instance, that they will ask about the convenience of the COVID vaccination. Some of their questions will be very easy to answer, but things will get complicated if they ask me about the future. For instance, Ana Isabel could ask: Can I stop my infliximab? And our response would, should, and will be: we do not know. An apparently simple question is not so simple. As we do love books, we will, first, quote some recent ones for establishing context.

First, communication between patients and physicians is not always easy (1). Making decisions is also complicated (2, 3). Besides, much medical advice does not resist the test of time (4). As humans, we have complex behaviors, sometimes “at our best” sometimes “at our worst” (5). We should be conscious of our limitations, and experts on Healthcare Systems have given us excellent guidelines to improve our systems (6, 7). In a world where artificial intelligence is taking the lead (8), “predicting and preempting disease” remains a very complex matter, as Eric Topol tells us in his provocative and inspiring books (9, 10). In the foreword of the last book, Abraham Verghese quotes this sentence from the Danish philosopher Soren Kierkegaard: “Life can only be understood backwards; but it must be lived forwards.” We cannot imagine a better description of our daily clinical task.

Coming back to IBD, two excellent recent reviews summarize the concept and possibilities of personalized medicine in Crohn's disease (11) and in IBD (12). Our goal in the present Research Topic is to help practical clinicians by providing some clues for prediction in some typical scenarios of an everyday IBD clinic: using biomarkers, microbiota clues, and responses to antiTNF, vedolizumab, and ustekinumab are some examples. For the exact question of my patient, we would need to study Edouard's views in his review (Louis), revisiting the clinical record of Ana Isabel, and being ready to listen to her opinion. Our current available tools can give estimates of “a risk of relapse of 25% in 3 years.” This data is of scientific interest, but is of a very relative value in a given person. Our ability to predict on an *individual* basis is poor, excepting very specific circumstances (11, 12). The fears, previous experiences, and very personal optics and circumstances of the patient will affect the conversation and the final decision (1). Of course, the current state of knowledge could change, and even be completely reverted (4). A patient's and physician's conversation will not be isolated from system and social circumstances (7). For instance, if a patient is under infliximab and azathioprine combination, a rather typical one in IBD, when considering withdrawal of one of them, efficacy and presumed toxicity should be the main issues, but insurers and payers will see price as a very important issue, and limit (sometimes decisively) the election of the cheapest one.

We think that the aspects we have discussed in this Research Topic are of maximal interest for IBD clinicians and patients. However, as should be the norm in good science, there remain more questions than answers. We would like to finish by asking for more investigator driven research, making randomized clinical trials with high ethical standards, the only way to make prediction easier and reversal rare (4). For Ana Isabel, the results of the SPARE trial are eagerly awaited.

## Author Contributions

All authors listed have made a substantial, direct and intellectual contribution to the work, and approved it for publication.

## Conflict of Interest

FG has received payments for talks and educational tasks from Janssen, Abbvie, MSD, and Takeda; and his group has received research grants from MSD, Abbvie, Janssen, Takeda, and Pfizer. The remaining author declares that the research was conducted in the absence of any commercial or financial relationships that could be construed as a potential conflict of interest.
